# Effects of vitamin D supplementation combined with resistance exercise on body composition and metabolic variables in older women: a randomized clinical trial

**DOI:** 10.1016/j.clinsp.2026.100942

**Published:** 2026-04-19

**Authors:** Illana Patricia da Silva Queiroz, Angélica Castilho Alonso, Vanderlei Carneiro da Silva, Adriana Machado-Lima, Priscila Long Amorim Bichara Pipa, José Maria Soares-Junior, Edmund Chada Baracat, Júlia Maria D’Andréa Greve, Marta Ferreira Bastos, Guilherme Carlos Brech

**Affiliations:** a Graduate Program in Aging Sciences, Universidade São Judas Tadeu, São Paulo, SP, Brazil; b Movement Study Laboratory, Institute of Orthopedics and Traumatology, Hospital das Clínicas da Faculdade de Medicina da Universidade de São Paulo (HC-FMUSP), São Paulo, SP, Brazil; c Department of Obstetrics and Gynecology, Gynecology Discipline, Hospital das Clínicas da Faculdade de Medicina da Universidade de São Paulo (HC-FMUSP), São Paulo, SP, Brazil; d Graduate Program in Physical Education, Universidade São Judas Tadeu, Brazil

**Keywords:** Aging, Vitamin D supplementation, Physical exercise, Body composition

## Abstract

**Introduction:**

Aging has a significant impact on health, leading to changes in body composition and physical functionality in older adults.

**Objective:**

Given the relevance of this topic, the aim of this study was to analyze the effects of vitamin D supplementation combined with physical exercise over a 12-week period on body composition and metabolic profile in elderly women.

**Methods:**

The study followed a clinical approach, employing a randomized, double-blind, placebo-controlled trial, in accordance with the guidelines established by the Consolidated Standards of Reporting Trials (CONSORT), conducted at the Movement Studies Laboratory in São Paulo, Brazil. Analyses were performed using an intention-to-treat approach with random effects regression models. The study sample consisted of 46 elderly individuals, divided into two groups with 23 participants each. The experimental group received vitamin D3 supplementation at a dose of 49,000 IU/week, while the placebo group received seven capsules of the same size, volume, and color, containing lactose but no vitamin D3.

**Results:**

After analyzing the results, it was observed that the groups were homogeneous in terms of age (p = 0.7658) and BMI (p = 0.5904). The results showed differences in 25-OH vitamin D levels (p < 0.001) between the groups over time.

**Conclusion:**

In conclusion, vitamin D supplementation successfully increased serum levels but failed to provide additional benefits for muscle mass or metabolic markers compared to resistance training alone.

## Highlights

• Vitamin D and exercise may benefit muscle health.
• Changes in body composition and bone health may require time.
• Vitamin D and exercise regulate biochemical parameters.

## Introduction

Aging is a natural, universal, and irreversible phenomenon that does not occur simultaneously or uniformly among individuals. Growing older is part of life, and in light of current scientific knowledge, there is nothing that can be done to alter this process. According to the Brazilian Institute of Geography and Statistics (IBGE), the elderly population in Brazil grew significantly, with 10.9% of people aged 65 or older and 15.8% aged 60 or older. The country's median age rose to 35, and the aging index, which measures the ratio of elderly people to children, reached 55.2 (for those 65+), indicating a demographic transition toward an older population.^1^

Aging brings various physiological changes, which, when combined with a sedentary lifestyle, lead to alterations in body composition, primarily an increase in abdominal fat and a decrease in muscle tissue. These changes become risk factors for low bone mineral density and predispose individuals to other disabilities.^2^

The aging process compromises the maintenance of the central nervous system, affecting physiological functions and balance control. These degenerative changes reduce the ability to adapt reflexively, which can result in dizziness, vertigo, and instability among the elderly, factors that significantly limit their quality of life.^3^ That balance issues can lead to decreased lower limb strength, contributing to instability and increasing the risk of falls and fractures.^4^

Genetic and biochemical factors associated with this stage of life result in cellular and tissue damage, mainly a reduction in muscle and bone tissue, which alters overall metabolism and contributes to increased body fat. Additionally, physiological changes occur in various systems, especially the endocrine system, when compared to younger organisms.^5^

Vitamin D supplementation has gained prominence, as the vitamin plays a crucial role in calcium absorption and bone health, and is also associated with muscle function. Sun exposure is one of the main sources of vitamin D, but the skin’s ability to produce this vitamin declines with aging. Vitamin D deficiency or insufficiency may contribute to the development of several conditions, such as cardiovascular, bone, cancerous, psychological, and immunomodulated diseases. Therefore, maintaining adequate vitamin D levels in the body supports overall health.^6^, ^7^, ^8^, ^9^

Observational studies also suggest a correlation between sufficient serum levels of vitamin D and a more favorable lipid profile. Individuals with adequate vitamin D levels have demonstrated a more positive lipid profile compared to those with deficiency or insufficiency.^10^^,^^11^

Given the importance of this vitamin to the human body, the present study aims to analyze the effects of vitamin D supplementation combined with physical exercise over 12-weeks on body composition in elderly women. The authors hypothesized that vitamin D supplementation would augment the effects of resistance exercise on lean mass and metabolic markers compared to exercise alone.

## Methods

### Study characteristics

The research is characterized as a randomized, placebo-controlled, double-blind clinical trial following the Consolidated Standards of Reporting Trials (CONSORT) guidelines.

The study was conducted by São Judas Tadeu University in conjunction with the Movement Studies Laboratory of the Institute of Orthopedics and Traumatology of the Hospital das Clínicas of the University of São Paulo School of Medicine and was approved by the Local Ethics Committee of the University of São Paulo under registration number 306/15 (Appendix 1), available at: Clinicaltrials.gov as NCT (03367685). All subjects signed the informed consent form.

### Sample group

The sample size for this study was determined based on the primary outcome of the parent trial (e.g., muscle strength or functional capacity), as previously described and published.^17^ Based on that calculation, 23 participants per group. The outcomes presented in the current manuscript are part of a secondary analysis of this larger trial.

The sample group was recruited through the dissemination of the research on social media such as Facebook and Instagram, where a registration form was made available. After the dissemination stage, a total of 422 elderly women were enrolled. The profile analysis process then began, in which the inclusion and exclusion criteria were established and identified through a clinical examination performed by a medical professional. The inclusion criteria were determined: Age over 60-years; Osteoporosis or osteopenia (bone mineral density; > -1.5 standard deviations from the T-score); Hypovitaminosis D (< 30 nmoL/L); Not exercising regularly; No injury or trauma to the lower limbs in the last three months that would prevent physical activity; Ability to walk independently and without limping for at least 100 meters; Independence in activities of daily living; Have no restrictions on resistance exercise, including having undergone a recent stress test (maximum 6-months); Not use medications such as estrogens or diuretics for bone mass gain; Not use dietary supplements containing vitamin D; Not have hyperparathyroidism, diabetes, uncontrolled hypertension, hyperprolactinemia, hypercalciuria, kidney stones, or high serum calcium, as previously assessed by a physician who will request the relevant tests for the inclusion of elderly women. The following items were established as exclusion criteria: Inability to perform any of the tests; Systolic blood pressure equal to or above 160 mmHg and diastolic blood pressure equal to or above 120 mmHg at the time of the test or during exercise; Missing more than three consecutive sessions during the intervention; Failure to attend the reassessment; Adverse reactions to the use of supplements.

After applying the criteria, a group of 46 elderly women participated in the study. The sample group also answered a questionnaire for the identification and collection of anthropometric data.

After collection, the allocation process for the groups was carried out using a randomized and stratified numerical sequence. The study consisted of two parallel arms, a control group (placebo) and an experimental group. Both groups performed the same physical exercises; however, one group was supplemented with vitamin D and the other received a placebo. In this study, participants were randomly assigned to either the treatment or control group using a stratified block randomization procedure. This sequence was kept confidential and stored in a location where the blinded evaluators did not have access.

### Evaluation

The evaluations were performed at two time points. Before starting the intervention (T1). After 12-weeks of intervention (T2). All physical evaluations and the intervention were performed at the Movement Study Laboratory of the Institute of Orthopedics and Traumatology of the Hospital das Clínicas of the Faculty of Medicine of the University of São Paulo (IOT-HC/FMSUP).

### Perimetry

All perimetry measurements were performed in a standardized manner, with three measurements taken to obtain the average of three measurements, using a non-elastic tape for all circumferences.

Triceps surae (calf): The calf Circumference (CC) was measured with a non-elastic tape with the elderly person in an upright position, with feet 20 cm apart, at the maximum circumference in the plane perpendicular to the longitudinal line of the calf.

Arm: The circumference of the contracted and flexed arm (CA) was determined by wrapping the measuring tape perpendicularly around the central region of the arm, aiming to achieve the largest measurement. The seated individual should keep the shoulder joint at 90° flexion in relation to the trunk and the elbow joint flexed, forming a 90° angle between the arm and forearm with the hand in the supine position.^13^ Still in the sitting position, the circumference of the leg (P) was determined by wrapping the measuring tape perpendicularly around the leg to obtain the largest value.

Thigh: The circumference of the thigh was measured in the middle third of the thigh, between the inguinal fold and the suprapatellar line, with participants standing upright and their feet flat on the floor.

### Body composition assessment

Percentage of fat (%); tissue (g); fat (g); lean mass (g), Bone Mineral Composition (BMC) (g), and Bone Mineral Density (BMD) (g/cm^2^) were assessed by bone densitometry using dual-energy X-Ray Absorptiometry (DXA) at LUNAR-DPX (Madison, Corporation, USA), performed at the Radiology Service of IOT-HC-FMUSP. The exams were performed by radiology technicians trained in the methodology and evaluated by the physician responsible for the service.

Total body mass and body composition were measured using an INBODY® model 230 multipolar bioimpedance scale. Patients were evaluated barefoot and wearing as little clothing as possible. Values related to age, height, and sex were entered into the scale.

### Laboratory analyses

Blood samples were collected from participants in tubes before and after the intervention. The following laboratory tests were analyzed: CPK, Glycated Hemoglobin (%), Glycated Hemoglobin, Non-HDL, VLDL, LDL, HDL, Potassium, Calcium, Phosphorus, Sodium, Cholesterol, Triglycerides, PTH, and 25OH Vitamin D. The blood was analyzed by the clinical analysis laboratory of HC/FMUSP.

### Intervention

The entire intervention was carried out at the Movement Study Laboratory of the Institute of Orthopedics and Traumatology in the Clinicas Hospital of the Medicine College in the University of São Paulo (IOT-HC/FMSUP).

### Physical activity

For the resistance exercise program, participants attended twice a week for twelve weeks. The program was designed to increase muscle mass. Each session was monitored by a blind researcher who was an exercise training specialist. The session lasted 45-minutes, with the first 5-minutes of walking to warm up the muscles, 25-minutes of strengthening for the lower limb muscles, 10-minutes for balance training, and the last 5-minutes of stretching for the muscles worked.

The resistance exercises were performed unilaterally (both legs) on mechanotherapy equipment (Biodelta Inc., São Paulo, Brazil). Three sets of 8‒12 repetitions were performed for each muscle group, with 30 to 60 seconds of rest between each set.

The participants underwent a 1-Max Rep (RM) test to measure and determine the amount of load applied to each exercise. The test refers to the maximum amount of weight that can be lifted in a single repetition of the proposed exercise. Three attempts were made to reach the plateau in the 1-RM score with a 3-minute interval between attempts.

The initial workload was set at 60% of the 1-RM maximum of the weaker leg. To promote sufficient workload and produce improvements over the 12-weeks of training, the exercise intensity was increased by 5%‒10% whenever the participants had adapted to the exercise workload, reaching a maximum of 80% of the maximum load.

After warming up with a 5-minute walk, muscle strength training began. The training focused on the lower limb muscles, due to their relationship with postural balance and falls.

The hip abductor muscles were worked with the participants in a lateral decubitus position on a stretcher or mat, with shin guards fixed to the ankle region and the contralateral lower limb supported in flexion to stabilize the trunk and avoid compensation when performing the exercise (SLR). The authors asked the patient to keep the lower limb extended and to increase the number of times proposed.

The hip adductor muscles were worked on an “adductor chair”, where the participants sat with their lower limbs abducted and performed the adduction movement against the resistance of the device, which had an independent lever for each limb and was overloaded by placing a weight plate on it.

The knee extensor muscles were worked on the horizontal leg press, with the participants sitting on the seat, with their feet fully engaged in the device, maintaining hip width, so that the plantar region remained in contact with the device. The authors asked them to push the footrest, thus extending their knees. This muscle group was also worked on the “extensor chair” with the participants seated, the front of their legs supported on the equipment, and they were asked to extend their lower limbs. This equipment has an independent lever for each limb, and overload was achieved by adding weights.

The triceps surae muscle was worked on the “leg press”, with the participants seated on the seat and asked to push the device with only the forefoot.

The knee flexor muscles, hamstrings, were worked on the “standing crural flexor” machine, with the participants standing, the front of the thigh resting on the machine, and the ankle supported, performing the knee flexion movement by pushing the lever with the load. This equipment also has an independent lever for each limb, and the overload was achieved by adding weight plates.

The last exercises for lower limb strength gain were squats. The participants remained standing with their torsos straight. The authors asked them to do a squat (up to 45° knee flexion) while holding a dumbbell. These exercises were always performed in front of a mirror to maintain the best posture.

For better posture maintenance in relation to postural balance. The authors did the pectoral muscle exercise on the “chest press” where the participants sat on a bench, maintaining a 90° abduction and flexion of the shoulder, and held the bar with their hands and were asked to push the bar by extending the elbow while maintaining 90% flexion and abduction of the shoulder. This equipment also has an independent lever for each limb, and overload is achieved by adding weights. In addition, some trunk maintenance muscles (trapezius, posterior deltoid, teres major, latissimus dorsi, biceps, and brachialis) were worked in the “row,” where participants sat on a base maintaining a semi-flexion of the lower limbs and performed the movement of pulling the bar while maintaining a 90° shoulder flexion. This equipment also has an independent lever for each limb and an overload made by placing a weight plate.

### Vitamin D3 supplementation

Supplementation was administered weekly to individuals who performed exercise training at the Movement Studies Laboratory, always at the same time, and ensuring that they ingested the appropriate amount. The experimental group took 49,000 IU/week of vitamin D3 supplementation. The placebo group ingested seven capsules of equal size, volume, and color, composed of lactose, without vitamin D3 supplementation.

### Statistical analysis

Quantitative variables are presented with mean, standard deviation, median, and interquartile range. Categorical variables are presented by frequency and proportion. The normal distribution of quantitative variables was assessed using histograms and the Shapiro-Wilk test. Comparisons of means and medians by group (control vs. supplementation) were assessed using the Student's *t*-test or Mann-Whitney test, when appropriate.

Associations between categorical variables were assessed using the Chi-Square test and Fisher's exact test. Correlations between biochemical and body composition parameters were evaluated using Pearson or Spearman correlations. To evaluate differences in biochemical and body composition parameters between groups, intention-to-treat analyses were performed with random effects regression models using SAS On Demand for Academics (Proc Mixed Procedure) software.

This type of analysis evaluates the rate of change in the outcome by interaction of treatment versus time and considers the response profile of correlations of repeated measures over time. The model incorporated terms of time, treatment group (supplementation), and time versus treatment group. A composite symmetry covariance pattern was used to construct the models, and time was included as a categorical variable to estimate the rate of change from baseline in biochemical and body composition parameters. The null hypothesis is that the mean response profiles are similar across groups. The significance level was set at p < 0.05 in all analyses.

## Results

In this study, initial clinical analysis showed that there was no statistical difference between the participants' body mass (kg), height (m), and BMI (kg/m^2^). The data characterizing the control and supplementation groups are presented in Table 1.

**Table 1:** Participant characteristics by group.

	Control (n = 23)				Supplementation (n = 23)				p-value
	Mean	SD	Median	IQR	Mean	SD	Median	IQR	
Age (years)	65.17	3.92	65	61.00‒69.00	65.78	4.57	64	62.00‒70.00	0.7658
Age menopause (years)	46.91	6.66	48	45.00‒52.00	48.17	5.53	48	46.00‒53.00	0.6045
Body Mass (Kg)	66.63	11.84	65	56.50‒73.00	62.6	9.15	63	55.60‒71.00	0.328
Height (m)	1.56	0.07	1.55	1.49‒1.61	1.55	0.66	1.54	1.51‒1.57	0.809
BMI (Kg/m^2^)	27.51	4.62	26.29	24.46‒28.95	25.95	3.16	26.22	23.68‒28.37	0.5904
Income	4.78	3.08	4	3.00‒6.00	4.26	3.27	3	2.00‒7.00	0.3408
Minutes of Physical Activity	67.61	113.44	0	0.00‒120.00	59.13	106.72	0	0.00‒90.00	0.8768

SD, Standard Deviation; IQR, Interquartile Range; kg, Kilograms; BMI, Body Mass Index.

*p-values derive from the Student's *t*-test or Mann-Whitney test.

Both sample groups reported that most of them did not experience hot flashes and were non-smokers. There was a higher proportion of married participants in the supplementation group. The categorical data characterizing the control and supplementation groups are presented in Table 2.

**Table 2:** Categorical data characterizing participants by groups.

	Control (n = 23)		Supplementation (n = 23)		p-value
	n	%	n	%	
**Feels Hot Flashes**					
No	21	91.3	18	78.26	
Yes	2	8.7	5	21.74	0.207
**Race**					
White	17	73.91	22	95.65	
Black	4	17.39	1	4.35	
Asian	2	8.7	-	-	0.111
**Education**					
Elementary	3	13.04	4	17.39	
High School	11	47.83	11	47.83	
College	9	34.78	8	34.78	1
**Marital Status**					
Married	7	30.43	17	73.91	
Divorced	7	30.43	2	8.7	
Separated	1	4.35	2	8.7	
Single	8	34.78	2	8.7	<0.05
**Smoking**					
No	22	100	22	95.65	
Yes	0	0	1	4.35	0.511
**Lives Alone**					
No	15	65.22	21	91.3	
Yes	8	34.78	2	8.7	<0.05

* p-values are derived from the Chi-Square test/Fisher's exact test.

Regarding the triceps surae, thigh, and arm circumference data, a difference can be observed only in relation to the left triceps surae at baseline, which was smaller in the supplemented group (Table 3).

**Table 3:** Participants' circumference data by group and period.

	Control (n = 23)				Supplementation (n = 23)				p-value
	Mean	SD	Median	IQR	Mean	SD	Mediana	IQR	
Triceps surae R before	37.23	4.24	37	34.00‒38.00	34.89	2.64	35.5	33.00‒36.50	0.089
Triceps surae R post	36.3	3.22	36	34.00‒38.00	35.39	5.11	35	33.00‒37.50	0.2521
Thigh R before	48.71	6.31	49	46.00‒53.00	47.28	3.84	48	45.00‒50.00	0.3568
Thigh R post	48.92	4.12	48	47.00‒51.00	46.21	5.71	47	44.00‒50.50	0.072
Arm R before	29.52	2.65	29	28.00‒31.00	28.86	3.27	27.8	27.00‒31.00	0.4563
Arm R post	29.02	2.86	29	27.00‒30.50	28.84	4.66	29	26.50‒30.00	0.5879
Triceps surae L before	37.43	4.27	37	34.00‒39.00	34.78	2.69	35	33.00‒37.00	**0.0401**
Triceps surae L post	36.65	3.03	36.5	34.50‒38.00	35.3	5.08	35.5	33.00‒38.00	0.1723
Thigh L before	48.69	6.25	49.5	45.50‒52.00	47.04	4.19	47.5	45.00‒51.00	0.2988
Thigh L post	48.78	4.15	48.5	45.50‒52.00	46.41	5.36	47	42.50‒51.00	0.1012
Arm L before	28.86	2.53	29	27.00‒31.00	28.61	4.57	28.5	26.00‒30.00	0.4163
Arm L post	28.86	2.52	28.5	27.00‒30.50	28.6	4.56	28	26.00‒30.00	0.3497

SD, Standard Deviation; IQR, Interquartile Range; R, Right; L, Left.

*p-values are derived from the Student's *t*-test or Mann-Whitney test.

The data from the analysis of the effect of supplementation, in the pre- and post-supplementation periods of the control and supplementation groups, regarding body composition data evaluated by DXA and biochemical parameters evaluated by blood tests, are presented in Table 4, Table 5. In general, only for potassium and 25OH Vitamin D was there a difference between the groups over time, with an increase in the values of these parameters in the supplemented group when compared to the control group at baseline.

**Table 4:** Comparison of body composition parameters between groups and time points.

	Pré				Pós				Effect of Supplementation		
	Control (n = 23)		Supple (n = 23)		Control (n = 23)		Supple (n = 23)		Treatment × Time	EP	p-value
	Mean	SD	Mean	SD	Mean	SD	Mean	SD	Interaction		
**Total Fat Mass**	25618.14	7403.86	23567.65	5287.59	24226.72	5496.37	23137.84	5392.3	288.4	368.53	0.4385
**Total Lean Mass**	39783.3	5233.2	37832.98	4987.96	39509.23	4417.96	38121.7	5050.94	-234.29	283.67	0.4137
**Total**	67106.32	11939.95	63009.97	9327.28	65423.1	8999.96	62853.85	9619.33	35.6	455.12	0.938
**Total Fat**	37.62	4.77	37.1	4.31	36.67	4.49	36.47	4.3	0.4	0.38	0.303
**Fat Mass R**	4581.3	1406.37	4378.5	1157.12	4446	1309.2	4370.04	1128.37	8.53	114.78	0.9411
**Lean Mass R**	6189.94	1003.24	5833.19	1012.97	6235.67	1069.35	5893.54	988.34	-83.07	101.71	0.4189
**Total R**	11068.04	2241.39	10493.37	1899.08	10978.75	2152.66	10548.34	1824.26	-76.29	175.26	0.6657
**Fat R**	40.75	5.91	41.4	5.59	40.05	5.82	41.05	5.73	0.23	0.66	0.7263
**Fat Mass L**	4469.63	1264.79	4256.4	1172.88	4256.04	1193.26	4232.05	1106.61	89.8	107.36	0.4078
**Lean Mass L**	6045.74	993.35	5705.72	1057.59	6038.95	843.3	5838.8	1007.2	18.09	91.33	0.844
**Total L**	10798.84	2076.56	10234.89	2023.49	10591.19	1859.32	10358.13	1915.46	106.25	160.2	0.511
**Fat L**	40.73	5.57	41.11	5.37	39.66	5.56	40.52	5.04	0.55	0.66	0.4128

SD, Standard Deviation; SE, Standard Error; D, Right; E, Left.

*p-values are derived from the *t*-Student or Mann-Whitney test.

**Table 5:** Biochemical parameters between groups and time points.

	Pré				Pós				Effect of Supplementation		
	Control (n = 23)		Supple (n = 23)		Control (n = 23)		Supple (n = 3)		Treatment × Time	EP	p-value
**CPK**	95.64	50.72	90.68	31.59	93.95	40.77	81.31	30.59	-4.07	12.06	0.7155
**Percent Glycated Hemoglobin**	5.99	1.2	5.7	0.46	5.96	1.32	5.72	0.61	-0.02	0.1	0.8184
**Glycated Hemoglobin**	125.31	34.41	117.18	13.27	124.22	37.78	117.72	17.63	-0.61	2.93	0.8354
**No HDL**	140.82	37.44	142.52	32.09	132.47	36.28	140.54	36.56	5.17	8.84	0.5618
**Potassium**	4.38	0.33	4.27	0.19	4.4	0.27	4.51	0.33	0.22	0.08	**0.0157**
**Cholesterol**	204.09	33.94	197.43	33.48	196.3	35.07	198.27	37.25	7.29	8.68	0.4057
**Triglycerides**	130	64.5	123.91	54.75	116.65	82.14	121.72	54.1	10.2	13.42	0.4514
**VLDL**	23.78	8.55	23.04	6.87	22	11.03	23.13	7.31	1.75	1.96	0.3771
**LDL**	117.04	33.64	119.47	29.04	110.47	29.79	117.45	31.08	3.45	7.63	0.6534
**HDL**	62.81	14.55	54.91	12.68	63.82	13.6	57.72	15.35	1.53	1.95	0.4373
**25 OH**	22.65	4.87	22.92	13.61	23.6	5.52	63.93	19.06	40.06	3.59	**<0.001**
**Ca**	9.39	0.43	9.31	0.36	9.27	0.37	9.32	0.26	0.12	0.11	0.252
**Phosphate**	3.53	3.55	3.25	0.43	3.47	0.43	3.37	0.43	0.159	0.12	0.178
**Sodium**	141.86	2.55	141.73	1.91	141	1.99	141.9	1.7	0.86	0.68	0.2088
**PTH**	51.85	18.07	48	16.76	52.17	18.88	42.41	10.78	-6.78	3.36	0.0505

SD, Standard Deviation; SE, Standard Error; D, Right; E, Left; CPK, Creatine Phosphokinase; VLDL, Very-Low-Density Lipoprotein; HDL, High-Density Lipoprotein; LDL, Low-Density Lipoprotein; 25OH: 25-Hydroxyvitamin D; Ca, Calcium; PTH, Parathormone.

*p-values are derived from the *t*-Student or Mann-Whitney test.

Additional correlations (Tables A and B) were performed to explore secondary relationships.

## Discussion

Aging is a process in which the body undergoes various metabolic and physical changes that can significantly influence health and well-being. In light of these metabolic changes, adequate levels of vitamin D are particularly important, as it is essential for proper calcium absorption, promoting bone health and preventing conditions such as osteoporosis. In addition, vitamin D plays an important role in muscle function, the immune system, and the regulation of inflammatory processes.

However, the aging process, associated with low physical activity and low sun exposure, causes a deficiency of this vitamin in the elderly, contributing to fractures and metabolic changes. In view of these deficiencies, the present study sought to analyze the effects of vitamin D supplementation associated with physical exercise for 12-weeks on body composition in elderly women. After applying the research, a difference was observed in the circumference of the triceps surae of the left leg, in addition to differences in levels of 25OH Vitamin D, which was supplemented in one of the groups.

Regarding biochemical parameters, a statistically significant increase in serum potassium was observed in the intervention group (p = 0.0157). However, the mean values remained strictly within the normal physiological range (4.27 to 4.51 mmoL/L). Given the lack of a clear mechanistic link in the current literature between vitamin D supplementation and serum potassium levels in this context, this finding should be interpreted with caution. It may represent an exploratory result without immediate clinical significance for muscle health or a consequence of multiple statistical comparisons.

In this regard, it is noted that the study did not identify statistical differences between sociodemographic and anthropometric characteristics. According to the World Health Organization (WHO), individuals with a BMI between 25 and 29.9 kg/m^2^ are diagnosed as overweight and may already have some damage from excess fat.^12^ The data from this study corroborate the data found in the study by Vásquez,^13^ in which it was identified that elderly individuals who did not practice physical activities had an overweight BMI (mean -26.62).

A positive point in this study is that most of the participants are non-smokers, which is essential for the development of the study, since smoking interferes with the absorption of nutrients and vitamins, including vitamin D.^14^

In the comparison between the control and supplementation groups at the two moments of the study, pre- and post-supplementation, in the analysis of the perimetry values, a difference was observed only in the left triceps surae at the pre-moment, with the control group presenting higher perimetry values. The lack of major changes in perimetry values between the control and supplementation groups at the post-intervention stage can be attributed to the short intervention period in the study or insufficient resistance training loads.

In many cases, the effects of an intervention may take longer to become observable, especially when it comes to physiological changes that require gradual adaptations in the body. After six months of regular physical activity that muscles begin to increase in size.^15^ Iida et al.^16^ found differences in perimetry values in older women with low BMD. The authors expected to find differences between the groups at different times, depending on the resistance exercise performed with the study participants.

Vitamin D plays an essential role in the health and well-being of the elderly, as it stands out as an essential nutrient for the maintenance of various vital functions. However, as the aging process occurs, levels of this vitamin often decline in the body, thus contributing to a series of metabolic changes.

Regarding biochemical parameters, the study showed that vitamin D supplementation was successful in increasing blood levels of 25OH and reducing PTH levels among participants who received supplementation. In addition, the results indicated that vitamin D supplementation had no impact on body composition. 12-weeks of multimodal exercise and vitamin D supplementation were insufficient to induce significant changes in body composition or BMD. These null findings suggest that for this specific population, a longer intervention period or a different dosing strategy might be required to observe therapeutic outcomes beyond pharmacokinetic repletion.

Vitamin D aids in the absorption of calcium and phosphorus, minerals essential for bone health. Vitamin D plays a critical role in preventing these conditions by strengthening bones and minimizing the risk of falls and injuries.^17^, ^18^, ^19^, ^20^, ^21^, ^22^

The data from this study also corroborate the meta-analysis by Siddiqee,^23^ which found that vitamin D supplementation has been shown to be effective in preventing bone density loss and reducing the risk of fractures in the elderly.

Furthermore, Caballero-García's research^24^ identified that vitamin D helped maintain muscle function and balance. This corroborates the data from this study. Several studies have shown that vitamin D supplementation can reduce the risk of falls in the elderly, especially those with vitamin D deficiency.^25^

Given the results observed, it is evident that the research has relevant clinical implications because, even in a short period of time, it was able to promote significant vitamin D supplementation in the body composition of elderly women. Thus, contributing to bone health, muscle strength, and metabolic function.

However, it is important to note that the study has some limitations that should be considered when interpreting its results. First, the 12-week intervention period may not have been sufficient to fully capture the long-term effects of vitamin D supplementation associated with physical exercise in elderly women. Significant changes in body composition and bone health may require a longer time to fully manifest. In addition, the study sample may not fully represent the diversity of the elderly population, as specific characteristics of the participants, such as health history, lifestyle, and genetic factors, may influence the response to the intervention. Also baseline difference was observed in the left triceps surae circumference (Table 3), this variation may still represent a potential confounding variable.

## Conclusion

In conclusion, while vitamin D supplementation effectively increased serum 25(OH)D levels, it did not lead to significant improvements in muscle mass or metabolic markers beyond the effects of resistance training alone.

## Credit authors’ statement

IPSQ; PLABP: Visualization, Investigation. IPSQ; VCS; PLABP: Conceptualization, Methodology, Software. IPSQ; VCS; BRECH, GC; ALONSO, AC: Data curation, Writing- Original draft preparation. JMDAG; AML; VCS; MFB; SOARES-JÚNIOR, JM; BARACAT, EC: Writing- Reviewing and Editing. AML; BRECH, GC; ALONSO, AC: Supervision.

## Data availability statement

The datasets generated and/or analyzed during the current study are available from the corresponding author upon reasonable request.

## Declaration of competing interest

The authors declare no conflicts of interest.

## Appendix Supplementary materials

Image, application 1
