# Supplementary material for: Effects of vitamin D supplementation combined with resistance exercise on body composition and metabolic variables in older women: a randomized clinical trial
Source: Clinics (Sao Paulo). 2026 Apr 19;81:100942. doi: 10.1016/j.clinsp.2026.100942 (PMC13101711; doi:10.1016/j.clinsp.2026.100942)
Supplement: Supplementary file 1 [file mmc1.docx]

**CLINICS-D-25-01708_ Complementary Results**

**Complementary Results**

**Table A** Correlations between biochemical parameters and body composition experimental group.

| **Variables** | **Pre** | **Post** | **Pre** | **Post** | **Pre** | **Post** |
| --- | --- | --- | --- | --- | --- | --- |
|  | **Lean Mass L** | **Lean Mass L** | **Lean Mass R** | **Mass Lean R** | **Lean Mass** | **Lean Mass** |
|  | ***r*** | ***r*** | ***r*** | ***r*** | ***r*** | ***r*** |
| Cholest | -0.1789 | -0.1083 | -0.2254 | -0.1519 | -0.1078 | -0.1308 |
| Trigl | 0.0405 | -0.1113 | 0.0968 | -0.0872 | 0.1186 | -0.0632 |
| LDL | -0.0193 | 0.0286 | -0.0816 | 0.0120 | 0.0672 | 0.0354 |
| HDL | **-0.5540** | -0.3599 | **-0.4996** | -0.3027 | **-0.5669** | -0.4232 |
| No HDL | 0.0138 | 0.0391 | -0.0331 | 0.0293 | 0.1142 | 0.0512 |
| VLDL | 0.0615 | -0.0871 | 0.1051 | -0.0515 | 0.1408 | -0.0394 |
| Hemo Perc | 0.3150 | 0.1822 | 0.3212 | 0.2246 | 0.3434 | 0.2057 |
| Hemo Glyc | 0.3150 | 0.1822 | 0.3212 | 0.2246 | 0.3434 | 0.2057 |
| Ca | -0.3164 | 0.0099 | -0.1589 | -0.0282 | -0.3531 | -0.0983 |
| Phosphato | 0.1096 | 0.1400 | 0.0431 | -0.0809 | 0.0258 | 0.0995 |
| PTH | 0.1346 | 0.0030 | 0.0772 | -0.1534 | 0.0940 | -0.0876 |
| Sodium | 0.0932 | 0.1464 | -0.0255 | 0.1375 | 0.0386 | 0.1562 |
| Potassium | -0.0090 | -0.2621 | -0.0171 | -0.2498 | -0.0406 | -0.3336 |
| **Variables** | **Pre** | **Post** | **Pre** | **Post** | **Pre** | **Post** |
|  | **Fat Mass L** | **Fat Mass L** | **Fat Mass R** | **Fat Mass R** | **Fat Mass** | **Fat Mass** |
|  | ***r*** | ***r*** | ***r*** | ***r*** | ***r*** | ***r*** |
| Cholest | 0.0440 | -0.0812 | 0.0158 | -0.1128 | 0.0420 | 0.1383 |
| Trigl | -0.2134 | -0.1188 | -0.2984 | -0.2075 | 0.1126 | 0.1504 |
| LDL | 0.1172 | 0.1407 | 0.0618 | 0.0527 | 0.1928 | 0.3913 |
| HDL | -0.1276 | -0.2078 | -0.0772 | -0.0663 | **-0.4338** | -0.3178 |
| No HDL | 0.0984 | 0.1272 | 0.0460 | 0.0293 | 0.2486 | 0.3747 |
| VLDL | -0.1954 | -0.0704 | -0.2822 | -0.1453 | 0.1369 | 0.2173 |
| Hemo Perc | -0.2706 | -0.3433 | -0.2996 | -0.2972 | -0.0210 | -0.1475 |
| Hemo Glyc | -0.2706 | -0.3433 | -0.2996 | -0.2972 | -0.0210 | -0.1475 |
| Ca | -0.1997 | -0.2287 | -0.2786 | -0.2813 | **-0.442** | -0.1807 |
| Phospha | -0.1591 | -0.3328 | -0.1487 | -0.4458 | -0.2722 | -0.4199 |
| PTH | 0.2360 | 0.0854 | 0.2048 | 0.0960 | 0.1262 | -0.2010 |
| Sodium | 0.3847 | -0.1276 | 0.3446 | -0.1562 | 0.2510 | -0.0187 |
| Potassium | 0.1706 | -0.0777 | 0.2177 | -0.0838 | 0.0677 | -0.3663 |

*r*, Correlation coefficient; R, Right; L, Left; CPK, Creatine Phosphokinase; VLDL, Very-Low-Density Lipoprotein; HDL, High-Density Lipoprotein; LDL, Low-Density Lipoprotein; 25OH, 25-Hydroxyvitamin D; Ca, Calcium; PTH, Parathormone.

*p-values derived from Pearson's and Spearman's correlation coefficient.

**Table B** Correlations of biochemical parameters with body composition control group.

| **Variables** | **Prée** | **Post** | **Pre** | **Post** | **Pre** | **Post** |
| --- | --- | --- | --- | --- | --- | --- |
|  | **Lean Mass L** | **Lean Mass L** | **Lean Mass R** | **Lean Mass R** | **Lean Mass** | **Lean Mass** |
|  | ***r*** | ***r*** | ***r*** | ***r*** | ***r*** | ***r*** |
| Cholest | -0.3649 | -0.3069 | -0.3553 | -0.3769 | **-0.4445** | -0.3979 |
| Trigl | 0.0217 | -0.1497 | -0.1225 | -0.2994 | 0.0257 | -0.1904 |
| LDL | -0.3673 | -0.2920 | -0.3767 | -0.3988 | -0.4088 | -0.4005 |
| HDL | -0.1757 | -0.2125 | -0.0944 | -0.0667 | -0.2305 | -0.0808 |
| No HDL | -0.2931 | -0.2927 | -0.3426 | **-0.4340** | -0.3480 | **-0.4295** |
| VLDL | -0.0099 | -0.1548 | -0.1460 | -0.3209 | -0.0005 | -0.2183 |
| Hemo Perc | 0.1262 | 0.0666 | 0.1074 | 0.0065 | 0.2348 | 0.0705 |
| Hemo Glyc | 0.1262 | 0.0666 | 0.1074 | 0.0065 | 0.2348 | 0.0705 |
| Ca | -0.2218 | -0.1191 | -0.2841 | -0.0975 | -0.1381 | -0.0612 |
| Phosph | -0.2283 | -0.1546 | -0.2306 | 0.0987 | -0.2767 | -0.0137 |
| PTH | 0.3516 | 0.3618 | 0.2769 | 0.3313 | 0.3685 | 0.2629 |
| Sodium | -0.2705 | -0.0483 | -0.2344 | -0.1201 | -0.2430 | -0.1258 |
| Potassium | -0.0378 | 0.1741 | -0.1430 | 0.2330 | -0.1161 | 0.1139 |
| **Variables** | **Pre** | **Post** | **Pre** | **Post** | **Pre** | **Post** |
|  | **Fat Mass L** | **Fat Mass L** | **Fat Mass R** | **Fat Mass R** | **Fat Mass** | **Fat Mass** |
|  | ***r*** | ***r*** | ***r*** | ***r*** | ***r*** | ***r*** |
| Cholest | -0.1017 | -0.1888 | -0.1627 | -0.2848 | -0.3118 | -0.3993 |
| Trigl | 0.0336 | 0.0463 | -0.0879 | 0.0864 | 0.3024 | 0.3288 |
| LDL | -0.0638 | -0.1305 | -0.1231 | -0.1870 | -0.2936 | -0.4197 |
| HDL | -0.2525 | -0.2182 | -0.1525 | -0.3296 | -0.3667 | -0.3833 |
| No HDL | -0.0217 | -0.1390 | -0.0875 | -0.1746 | -0.1903 | -0.3526 |
| VLDL | 0.0287 | 0.0816 | -0.1020 | 0.1224 | 0.2569 | 0.2851 |
| Hemo Perc | -0.1131 | 0.0274 | -0.1762 | -0.1280 | 0.0222 | 0.1117 |
| Hemo Glyc | -0.1131 | 0.0274 | -0.1762 | -0.1280 | 0.0222 | 0.1117 |
| Ca | **-0.4441** | **-0.4831** | **-0.4640** | -0.4140 | -0.1111 | -0.2087 |
| Phosph | **-0.5614** | -0.3698 | **-0.4634** | -0.1923 | **-0.4526** | -0.2876 |
| PTH | 0.1540 | 0.3092 | 0.2957 | 0.2035 | 0.2385 | 0.3132 |
| Sodium | -0.2410 | -0.0534 | -0.3034 | 0.0695 | -0.3854 | -0.1253 |
| Potassium | -0.2861 | -0.0746 | -0.1585 | -0.1852 | -0.2655 | -0.1800 |

*r*, Correlation coefficient; R, Right; L, Left; CPK, Creatine Phosphokinase; VLDL, Very-Low-Density Lipoprotein; HDL, High-Density Lipoprotein; LDL, Low-Density Lipoprotein; 25OH, 25-Hydroxyvitamin D; Ca, Calcium; PTH, Parathormone.

*p-values derived from Pearson's and Spearman's correlation coefficient.

The correlation of biochemical parameters with body composition in the supplementation group is shown in Figure 1.

**Figure 1** Correlation of biochemical parameters with body composition in the supplementation group.

The correlation of biochemical parameters with body composition in the control group is shown in Figure 2.

**Figure 2** Correlation of biochemical parameters with body composition in the control group.
